# Throat and Lungs

**Published:** 1876-05

**Authors:** 


### THROAT AND LUNGS.

In a practice of seventeen years, devoted exclusively to the treatment of throat and lung affections, we have arrived at the following conclusions, that:

First: Throat-ail, or Clergymen's sore throat, called chronic laryngitis, is, in four cases out of five, originated in the stomach, and that to attempt to remove it by any other means than such as are adapted to improving the digestion and waking up the activities of the liver, is the sorriest absurdity of the age.

Second: When consumption of the lungs is threatened, or is actually present, the first great and efficient remedial agent, worth incomparably more than all the drugs on earth, is the spending of every hour of daylight possible, in the open air, in some moderate, unfatiguing employment, and the eating of as much plain, nourishing, and relished food as the stomach will digest. Next to that, as being more universally accessible, is an India-rubber Life Preserver, and for reasons which no physiologist of even ordinary acquirements would for a moment dispute.

The health of a man's lungs in reference to consumption, depends upon their capacity to receive the air he breathes. Hence that capability is called "vital capacity," and is measured by the amount of air the lungs can throw out at a full expiration. This capacity varies according to age, sex, weight, and stature; all of these can be safely left out of view in ordinary cases, except the hight. One man can blow up a bladder, can fill it at a breath; another in equal health of lungs would require two breaths, showing that the lungs of the former had twice as much air as those of the latter. The cubic method is that adopted for the measurement of the air in the lungs, or by the pint; and it can be as accurately done as if it were water, to the fraction of a gill or inch.

Forty cubic inches make a pint: a man of ordinary size, in good health of lungs, will expire at a single effort, six pints of air, or two hundred and forty cubic inches.

If a man five feet ten inches high could distend fully at a single breath, an India-rubber bag, bladder, or other receptacle, which held two hundred and forty cubic inches of air, it would be a physical demonstration, that all his lungs were within him, that they were in full operation, and as a matter of course, there could not possibly be, under the circumstances, any actual consumption, which would be corroborated beyond all cavil, if the pulse was uniformly under seventy beats in a minute.

A person never becomes consumptive until for many weeks, and for months, the lungs have worked imperfectly; thus working imperfectly, the system receives at each breath, less air than it requires; the blood is that much less purified; the body is that much less nourished; hence, as a man falls more and more decidedly into consumption, he has less breath, less blood, less flesh, less strength; this, all know.

But suppose a patient becomes acquainted with the fact that his lungs are declining in their capability of receiving air, losing their vital capacity, the evident indication would be to arrest that decline, and not rest satisfied until it was fully removed. And what more rational course than to practice on the lungs; to exercise them artificially; to accustom himself several times a day to blow upon his India-rubber; to try more and more on each occasion to fill it more fully at a single breath?

Some months ago a man came to us who could expire with the utmost effort only ninety-four inches; we sat him down among the incurables; we adjudged him to certain death; still he was urged to try. He promised he would. Ten days ago, March 17th, he presented himself again, having practiced these artificial breathings, and gave a measurement of a hundred and forty-four. Perseverance and an equal rate of increase for a few months longer, will certainly restore him. But this is only one of a multitude of similar cases.

The lesson of the article is:

If coming consumption is always attended with a diminution of vital capacity, of lung activity, of capability of full, free breathing, it must be averted by such practices as will arrest that decline first, and then reëstablish the activities. But nobody will heed these momentous lessons, because their practice would cost no money, and they have not the charm of mystery, nor the prestige of brazen trumpets and shameless falsehoods; hence we are not afraid of our practice declining by communicating the information, for we have done it for years, yet our report is as practically unbelieved as that of the prophet of the olden times.

